# Synthesis of oxa-bridged derivatives from Diels–Alder bis-adducts of butadiene and 1,2,3,4-tetrahalo-5,5-dimethoxycyclopentadiene

**DOI:** 10.3762/bjoc.6.64

**Published:** 2010-06-14

**Authors:** Faiz Ahmed Khan, Karuppasamy Parasuraman

**Affiliations:** 1 Department of Chemistry, Indian Institute of Technology, Kanpur-208 016, India

**Keywords:** Diels–Alder reactions, diketones, oxa-bridged derivatives, ruthenium, 3-sulfolene

## Abstract

Bis-adducts of 1,2,3,4-tetrahalo-5,5-dimethoxycyclopentadiene and 1,3-butadiene, generated in situ from 3-sulfolene, have been synthesized in excellent yield. Ruthenium catalyzed oxidation of the bis-adducts followed by a one-pot transformation of the resulting α-diketone furnished oxa-bridged compounds. Unambiguous stereochemical assignments of both diastereomeric series are reported.

## Introduction

**Scheme 1:**
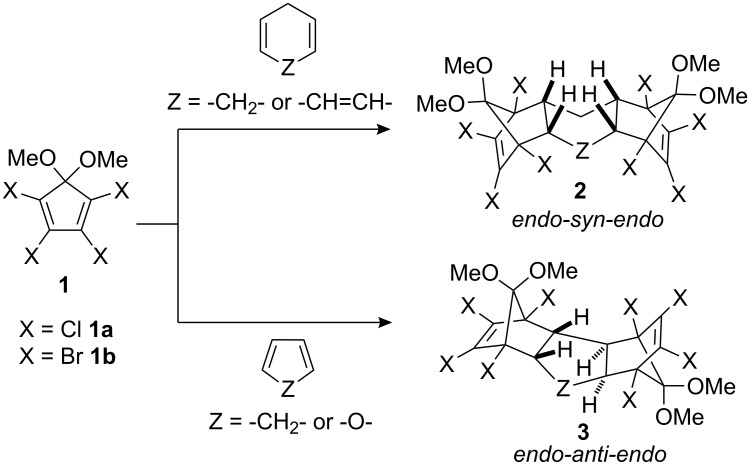
Diels–Alder bis-adducts of **1** with cyclic dienes.

3-Sulfolene is a nonflammable, nontoxic, nonhygroscopic and stable crystalline solid and is a convenient equivalent for gaseous 1,3-butadiene [1–3] and is commonly used for in situ generation of 1,3-butadiene as the diene component in Diels–Alder reactions. We and other groups have demonstrated the utility of cyclic dienes for the synthesis of 2:1 Diels–Alder bis-adducts with 1,2,3,4-tetrahalo-5,5-dimethoxycyclopentadiene **1** [4–7]. In the case of cyclic dienes (or trienes) such as cyclohexa-1,4-diene and cycloheptatriene, *endo-syn-endo* diastereomer **2** is formed exclusively, whilst cyclopentadiene and furan yield solely *endo-anti-endo* diastereomer **3** (Scheme 1). In continuation of our interest in the Diels–Alder bis-adducts of 1,2,3,4-tetrahalo-5,5-dimethoxycyclopentadienes **1** and their applications [8–14], we envisaged employing 1,3-butadiene as bis-dienophile component. Herein we report the synthesis of bis-adducts of 1,2,3,4-tetrahalo-5,5-dimethoxycyclopentadiene and butadiene followed by their transformation to oxa-bridged compounds. The stereochemistry of the diastereomeric products was also unequivocally established.

We were interested in exploring the previously overlooked stereochemical outcome of the Diels–Alder reaction between **1a** and 1,3-butadiene [15–16]. The bis-adduct obtained from **1a** and gaseous 1,3-butadiene was previously assigned as “*endo*, *exo*-bis(7,7-dimethoxy-1,2,3,4-tetrachloronorborn-2-en-5-yl)” [16]. In our reinvestigation we used 3-sulfolene as a 1,3-butadiene source to prepare both the mono- and bis-adducts. The two diastereomeric bis-adducts were separated and the relative stereochemistry was established by single crystal X-ray diffraction and ^1^H NMR spectroscopy. The bis-adducts were further transformed into bis-diketones by means of supported ruthenium catalyzed oxidation. Finally, the two diastereomeric norbornyl α-diketones from the chloro as well as the bromo series were each converted to the corresponding oxa-bridged compounds [7].

## Results and Discussion

**Scheme 2:**
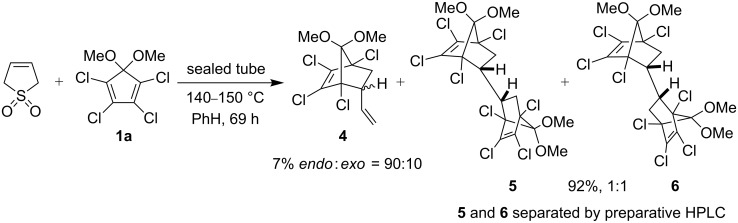
Diels–Alder reaction of **1a** with 3-sulfolene.

For the preparation of the 2:1 adducts, 2 equivalents of 1,2,3,4-tetrachlorodimethoxycyclopentadiene **1a** and one equivalent of 3-sulfolene were heated at 140–150 °C for 69 h in a sealed tube. The reaction mixture was purified by silica gel chromatography to afford the mono-adduct **4** in 7% yield as an inseparable mixture of *endo* and *exo* isomers [16] (*endo*:*exo* = 90:10, as determined by ^1^H NMR spectroscopy) and the two diastereomeric bis-adducts **5** and **6** as a 1:1 mixture in 92% yield (Scheme 2).

The assignment for the *exo*-isomer **4** is based on the H_5_-*endo* methine signal at 2.48 ppm which appears as a triplet of doublets. The corresponding H_5_-*exo* methine proton for *endo*-isomer **4** appeared at 3.2 ppm. The bis-adducts **5** and **6** were successfully separated by preparative HPLC [17]. Adduct **5**, a colourless crystalline compound with melting point 176–178 °C, displayed two singlets at 3.54 and 3.51 ppm for the methoxy groups, a multiplet at 2.45–2.42 ppm for two methine protons and another multiplet at 2.37–2.31 ppm for four methylene protons in its ^1^H NMR spectrum. In the ^13^C NMR spectrum, the methine carbon atoms appeared at 47.6 ppm, and the methylene carbon atoms at 41.4 ppm. By contrast, the diastereomer **6**, a colorless solid with melting point 182–184 °C showed two singlets at 3.57 and 3.50 ppm for methoxy groups, a doublet of doublets at 2.96 ppm for methine protons and two doublets of doublets at 2.33 and 1.34 ppm for the methylene protons in its ^1^H NMR spectrum. In the ^13^C NMR spectrum of **6**, the methine carbon atoms appeared at 43.7 ppm and the methylene carbons at 35.9 ppm.

**Scheme 3:**
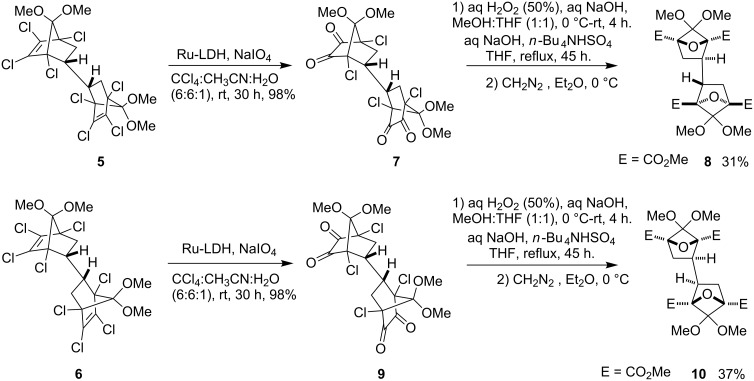
Synthesis of bis-oxa-bridged compounds **8** and **10** from bis-adducts **5** and **6**.

The bis-adducts **5** and **6** were smoothly transformed to the corresponding bis-α-diketones **7** and **9** in excellent yield with a supported ruthenium catalyst (Ru-LDH) and NaIO_4_ as stoichiometric co-oxidant, a methodology developed in our laboratory [18–19]. Previously, we reported a smooth one-pot transformation of norbornyl α-diketones to the corresponding oxa-bridged derivatives [7], but our initial attempts to transform the bis-diketones **7** and **9** to bis-oxa-bridged compounds **8** and **10** using this strategy did not give the desired result. However, when the reaction was carried out in presence of the phase transfer catalyst TBHSO_4_ the bis-oxa-bridged compounds **8** and **10** were obtained (after esterification with diazomethane) in 31 and 37%, respectively (Scheme 3).

**Figure 1:**
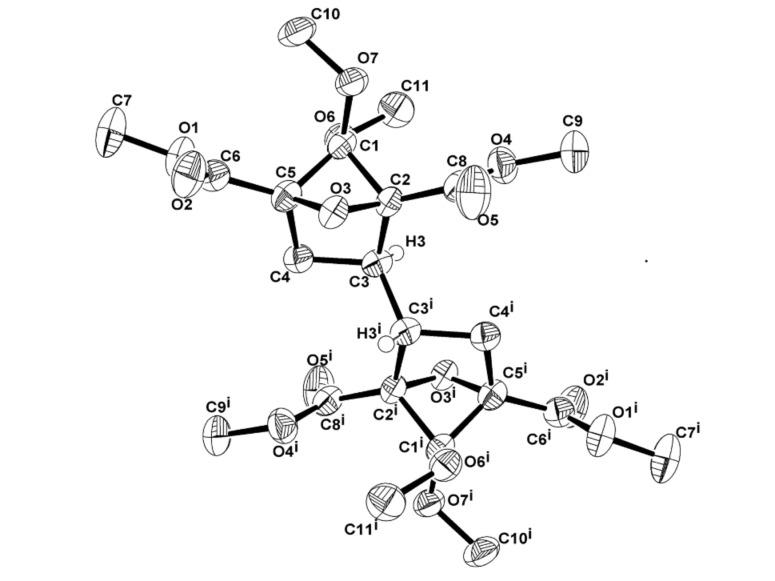
ORTEP structure of **8** [50% probability thermal ellipsoids; some of the hydrogen atoms and a solvent molecule (acetonitrile) are omitted for clarity].

The relative stereochemistry in **8** was unambiguously established by the single crystal X-ray analysis (Figure 1) [20]. Working backwards, the structures of the adduct **5**, the bis-diketone **7** were confirmed unequivocally.

**Scheme 4:**
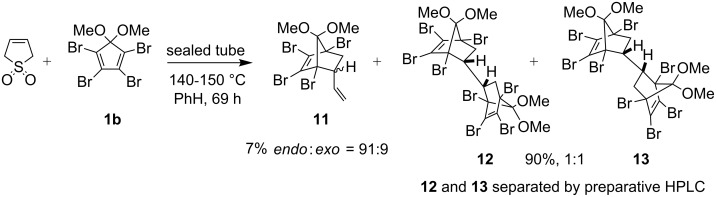
Diels–Alder reaction of **1b** with 3-sulfolene.

We next turned our attention to the bromo analogue **1b** in order to see if the overall yield of the bis-oxa-bridged derivatives **8** and **10** could be improved. We were also interested to see if any bromo derivative, corresponding to the diastereomer **6** in the chloro series, would furnish crystals suitable for X-ray analysis. The Diels–Alder reaction between 1,2,3,4-tetrabromo-5,5-dimethoxycyclopentadiene **1b** and 3-sulfolene under the same experimental conditions as described for the chloro-analogue furnished mono-adduct **11** (*endo*:*exo* = 91:9) and bis-adducts **12** and **13** (Scheme 4). The bis-adducts **12** and **13** were separated by preparative HPLC.

**Scheme 5:**
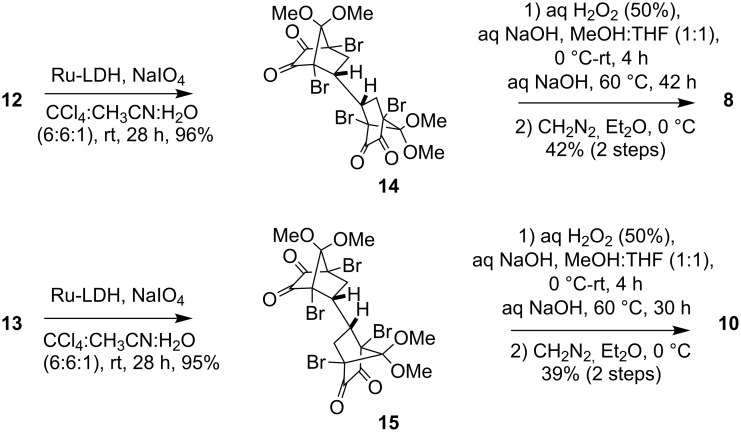
Synthesis of bis-oxa-bridged compounds **8** and **10** from bis-diketones **14** and **15**.

The bis-adducts **12** and **13** were converted in excellent yields to the corresponding bis-α-diketones **14** and **15** (Scheme 5). Bis-diketone **14** was treated first with alkaline H_2_O_2_ and then with additional NaOH (60 equiv) at 60 °C followed by esterification with diazomethane to obtain the oxa-bridged compound **8** in 42% yield. Bis-diketone **15** was transformed into **10** in 39% yield by a similar method. Unlike the bis-diketones in chloro series (**7** and **9**), which required a phase transfer reagent (TBHSO_4_), the bromo bis-diketones **14** and **15** underwent transformation to the bis-oxa-briged derivative **8** and **10** under the usual procedure previously reported from our laboratory [7] (Scheme 5). Although the yields in the final step were moderate (42 and 39%), this corresponds to 63–65% per oxa-bridge formed which is gratifying considering the number of intermediates involved and possible side reactions.

**Figure 2:**
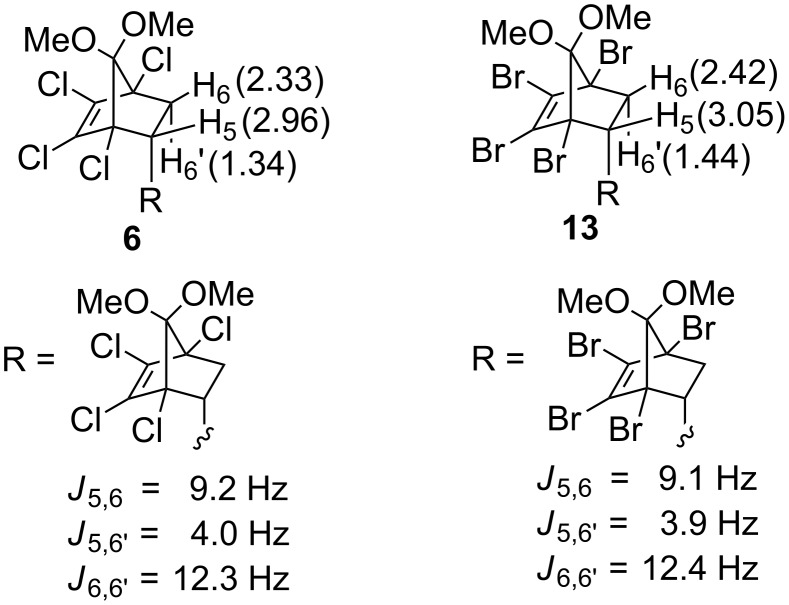
^1^H NMR chemical shifts (in parentheses) and coupling constants (*J*) for the three interacting protons (H_5_, H_6_, and H_6’_ ; for the sake of convenience, numbering sequence of mono-adducts is adopted) of the bis-adducts **6** and **13**.

Unfortunately, neither **13** nor **15** gave crystals suitable for X-ray analysis. However, unambiguous assignment was possible from the diagnostic chemical shifts and coupling constants observed for methine (H_5_) and methylene (H_6_ and H_6’_) protons of bis-adducts **6** and **13** (Figure 2). The appearance of H_5_ at ~3 ppm with characteristic coupling constants of ~9 and ~4 Hz to H_6_ and H_6’_, respectively, unequivocally supports the assigned structures. These values are consistent with several *endo*-substituted derivatives (R = alkyl-like groups) reported by us [9] and others [21–22]. The observed selectivity is in agreement with the strong *endo*-selectivity displayed by diene **1**.

**Figure 3:**
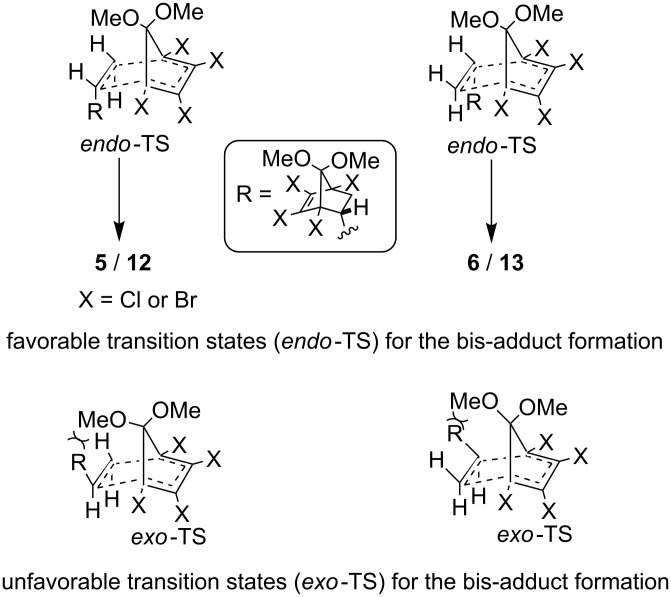
Transition state models for the bis-adduct formation.

From the above results it is clear that the diastereomeric bis-adducts **5**, **6** and **12**, **13** are formed via *endo*-*endo* addition. The proposed transition states for the formation of bis-adducts are shown in Figure 3. The initial *endo*-mono adduct (**4** or **11**) gives rise to two possible *endo*-transition states leading to **5**, **6** or **12**, **13**. The corresponding *exo*-transition states suffer from severe steric congestion due to the bulky R group and are consequently unfavorable. Similar steric considerations rule out the participation of an initially formed minor *exo*-mono adduct (**4** or **11**) to participate further in the reaction to give bis-adducts, thus ruling out the formation of diastereomers via *exo*-*endo* addition.

## Conclusion

In conclusion, we have demonstrated that the Diels–Alder reaction between **1** (diene component) and 1,3-butadiene (bis-dienophile component) proceeds via *endo*-*endo* addition mode to give a 1:1 mixture of diastereomeric bis-adducts. The diastereomeric bis-adducts were separated and transformed into bis-oxa-bridged compounds. The relative stereochemistry of the products was unambiguously established by single crystal X-ray diffraction and NMR spectroscopy.

## Supporting Information

File 1 General methods, experimental procedures and analytical data for new compounds.
