# Supplementary material for: Synthesis of oxa-bridged derivatives from Diels–Alder bis-adducts of butadiene and 1,2,3,4-tetrahalo-5,5-dimethoxycyclopentadiene
Source: Beilstein J Org Chem. 2010 Jun 14;6:64. doi: 10.3762/bjoc.6.64 (PMC2919264; doi:10.3762/bjoc.6.64)
Supplement: File 1 — General methods, experimental procedures and analytical data for new compounds. [file Beilstein_J_Org_Chem-06-64-s001.pdf]

Supporting Information

for

Synthesis of oxa-bridged derivatives from Diels–Alder bis-adducts of butadiene and 1,2,3,4-tetrahalo-5,5-dimethoxycyclopentadiene

Faiz Ahmed Khan* and Karuppasamy Parasuraman

Department of Chemistry, Indian Institute of Technology, Kanpur-208 016, India

Email: Faiz Ahmed Khan - faiz@iitk.ac.in

*Corresponding author

General methods, experimental procedures and analytical data for new compounds.

Contents

General Methods **S2**

Experimental Procedures and Analytical Data **S2**

Copies of ^{1}H and ^{13}C spectra **S7**

General Methods

All the reactions were performed in oven dried apparatus and the reaction mixtures were stirred magnetically. Thin layer chromatography was performed on silica gel coated on microscope slides. Visualization of spots was effected by exposure to iodine or spraying with 4% ethanolic H_2SO_4 and charring. Column chromatography was carried out with silica gel (100-200 mesh), with ethyl acetate-hexane as eluent unless otherwise noted. Evaporation of solvent was performed at reduced pressure on a rotary evaporator. Melting points are uncorrected. IR spectra were recorded as KBr pellets (solids) or thin films (liquids). ^{1}H NMR and proton-decoupled ^{13}C NMR spectra were recorded at 400 and 100 MHz, respectively on a JEOL spectrophotometer. NMR measurements were made in $CDCl_3$ or DMSO-d_6 with tetramethylsilane as internal standard. Solvents and reagent were purified by standard methods [1]. Elemental analyses were performed using a CE-440 Elemental Analyzer of Exeter Analytical Inc. HRMS spectra were recorded on a WATERS Q-TOF micro mass spectrometer with electron spray ionization (ESI). X-ray data was collected on a Bruker SMART APEX diffractometer. The structure was solved using SIR-92 and refined using SHELXL-97 [2].

Experimental Procedures and Analytical Data

Preparation of compounds 4–6

In a sealed tube a mixture of 3-sulfolene (446 mg, 3.78 mmol), 1,2,3,4-tetrachloro-5,5-dimethoxycyclopentadiene (2 g, 7.57 mmol), hydroquinone (2 mg) and epichlorohydrin (0.1 mL) in benzene (2 mL) was heated at 140–150 °C for 69 h. The volatiles were removed in vacuo and the products purified by silica gel column chromatography using 2–5% ethyl acetate/hexane as eluent to afford 105 mg of the mono adduct **4** (84 mg, 7%) as an *endo*:*exo* mixture (ratio 90:10) and the bis-adducts **5** and **6** as mixture of diastereomers in 1:1 ratio (2.02 g, 92%). The mixture **5** and **6** was quantitatively separated by preparative HPLC on a JAI LC-908W equipped with a JAIGEL-OA4100 column with 1% THF/hexane as eluent.

1,2,3,4-tetrachloro-7,7-dimethoxy-5-vinylbicyclo[2.2.1]hept-2-ene (4)

Colorless liquid; *endo*:*exo* = 90:10. IR (neat): 2800, 1600, 1440, 1260, 1180 cm^{-1}; ^{1}H NMR (400 MHz, $CDCl_3$): *endo* (major isomer): δ 5.52–5.43 (m, 1 H), 5.26–5.12 (m, 2 H), 3.62 (s, 3 H), 3.56 (s, 3 H), 3.2 (td, J = 8.8 Hz, 4.1 Hz, 1 H), 2.55 (dd, J = 11.8 Hz, 8.8 Hz, 1 H), 1.73 (dd, J =

11.8 Hz, 4.1 Hz, 1 H). *exo* (minor isomer): δ 6.04–5.95 (m, 1 H), 5.26–5.12 (m, 2 H, merged with major isomer), 3.62 (s, 3 H, merged with major isomer), 3.58 (s, 3 H), 2.48 (td, J = 8.8, 5.8 Hz, 1 H), 2.24–2.20 (m, 2 H); ^{13}C NMR (100 MHz, $CDCl_3$): *endo* (major isomer): δ 133.8, 130, 128.5, 119.5, 111.5, 78.6, 74.5, 52.6, 51.5, 50.9, 41.2; *exo* (minor isomer): δ 135.7, 130, 128.5, 120.1, 111.5, 78.6, 74.5, 52.4, 51.4, 51.2, 41.2; HRMS (ESI): *m/z* $(M+Na)^+$ calculated for $C_{11}H_{12}Cl_4O_2$: 338.9489; Found: 338.9489.

1,1',4,4',5,5',6,6'-octachloro-7,7,7',7'-tetramethoxy-2,2'-bi(bicyclo[2.2.1]heptane)-5,5'-diene (5)

Colorless solid; mp: 176–178 °C. IR (KBr): 2900, 1600, 1440, 1260, 1180 cm^{-1}; ^{1}H NMR (400 MHz, $CDCl_3$): δ 3.54 (s, 6 H), 3.51 (s, 6 H), 2.45–2.42 (m, 2 H), 2.37–2.31 (m, 4 H); ^{13}C NMR (100 MHz, CDCl3): δ 131 (2C), 127.6 (2C), 110.6 (2C), 79.4 (2C), 73.8 (2C), 52.7 (2C), 51.7 (2C), 47.6 (2C) 41.4 (2C); Anal. Calcd. for $C_{18}H_{18}Cl_8O_4$: C 37.15, H 3.12; Found: C 37.09, H 3.03.

1,1',4,4',5,5',6,6'-octachloro-7,7,7',7'-tetramethoxy-2,2'-bi(bicyclo[2.2.1]heptane)-5,5'-diene (6)

Colorless solid; mp: 182–184 °C. IR (KBr): 2900, 1600, 1440, 1260, 1180 cm^{-1}; ^{1}H NMR (400 MHz, $CDCl_3$,): δ 3.57 (s, 6 H), 3.5 (s, 6 H), 2.96 (dd, J = 9.2 Hz, 4.0 Hz, 2 H), 2.33 (dd, J = 12.3 Hz, 9.2 Hz, 2 H), 1.34 (dd, J = 12.3 Hz, 4.0 Hz, 2 H); ^{13}C NMR (100 MHz, $CDCl_3$): δ 129.5 (2 C), 128 (2 C), 111.5 (2 C), 78.7 (2 C), 74.4 (2 C), 52.7 (2 C), 51.7 (2 C), 43.7 (2 C), 35.9 (2 C); Anal. Calcd. for $C_{18}H_{18}Cl_8O_4$: C 37.15, H 3.12; Found: C 37.18, H 3.15.

1,1',4,4'-tetrachloro-7,7,7',7'-tetramethoxy-2,2'-bi(bicyclo[2.2.1]heptane)-5,5',6,6'-tetrone (7)

To stirred solution of bis-adduct **5** (251 mg, 0.43 mmol) in acetonitrile (3 mL) and carbon tetrachloride (3 mL) at room temperature, was added supported ruthenium catalyst (Ru-LDH, 35 mg), $NaIO_4$ (369 mg, 1.72 mmol) and distilled water (0.4 mL). The reaction mixture was stirred for 30 h, then filtered through a small pad of silica which was washed with dichloromethane (15 mL) and the filtrate concentrated. The residue was dissolved in ethyl acetate (10 mL) and washed with aqueous sodium thiosulfate solution (2 x 2 mL). The organic layer was dried over anhydrous sodium sulfate, filtered and concentrated under vacuum to afford 213 mg of the bis-diketone **7**. Yield: 98%.

Yellowish solid; mp: 230 °C (decomp.). IR (KBr): 2900, 1760, 1440, 1180 cm^{-1}; ^{1}H NMR (400 MHz, DMSO-d_6,): δ 3.44 (s, 6 H), 3.4 (s, 6 H), 2.86 (dd, J = 12.2 Hz, 1.7 Hz, 2 H), 2.29–2.27 (m, 2 H), 2.08–2.02 (m, 2 H); ^{13}C NMR (100 MHz, DMSO-d_6): δ 200.9 (2 C), 102.2 (2 C), 94.7 (2 C), 80.6 (2 C), 73.3 (2 C), 51.8 (2 C), 51.1 (2 C), 41.1 (2 C), 36.2 (2 C); HRMS (ESI): *m*/*z* $(M+H)^+$ calculated for $C_{18}H_{18}Cl_4O_8$: 502.9835; Found: 502.9835.

Preparation of bis-oxa-bridged compound 8

To a stirred solution of bis-diketone **7** (150 mg, 0.297 mmol) in MeOH (4mL) and THF (4 mL), was added 50% H_2O_2 (0.07 mL) followed by 6N NaOH (0.3 mL) at 0–5 °C. The reaction mixture was stirred for 4 h at room temperature and the solvents removed in vacuo. To the residue, THF (4 mL), tetrabutylammonium hydrogen sulfate (222 mg, 0.654 mmol) and 50% NaOH solution (2.5 mL) were added at 0–5 °C. The reaction mixture was heated at reflux for 45 h and the mixture then acidified with 10% HCl (pH = 3) at 0–5 °C. The solution was extracted with ethyl acetate (4 x 4 mL), the combined organic layers washed with brine (2 mL) and dried over anhydrous sodium sulfate. The crude carboxylic acid obtained on concentration was treated with excess diazomethane in ether/methanol (1:1) at 0 °C. The oxa-bridged compound **8** was obtained after silica gel column chromatography using 50% ethyl acetate / hexane. Yield: 31%.

Colorless solid; mp: 234 °C (decomp.). IR (KBr): 2900, 1740, 1430, 1200 cm^{-1}; ^{1}H NMR (400 MHz, $CDCl_3$,): δ 3.8 (s, 6 H), 3.79 (s, 6 H), 3.43 (s, 6 H), 3.3 (s, 6 H), 2.95–2.94 (m, 2 H), 2.49–2.45 (m, 2 H), 1.7 (dd, J = 11.0 Hz, 2.2 Hz, 2 H); ^{13}C NMR (100 MHz, $CDCl_3$): δ 166.9 (2 C), 166.7 (2 C), 109.8 (2 C), 92.2 (2 C), 88.2 (2 C), 52.7 (2 C), 52.6 (2 C), 51.98 (2 C), 51.94 (2 C), 43.6 (2 C), 35.9 (2 C); HRMS (ESI): *m*/*z* $(M+H)^+$ calculated for $C_{22}H_{30}O_{14}$: 519.1715; Found: 519.1716.

1,1',4,4'-tetrachloro-7,7,7',7'-tetramethoxy-2,2'-bi(bicyclo[2.2.1]heptane)-5,5',6,6'-tetrone (9)

The reaction was performed as described for **7**; yield: 98%; yellowish solid; mp: 230 °C (decomp.). IR (KBr): 2900, 1760, 1440, 1180 cm^{-1}; ^{1}H NMR (400 MHz, DMSO-d_6,): δ 3.45 (s, 6 H) 3.41 (s, 6 H), 2.72 (dd, J = 12.6 Hz, 4.6 Hz, 2 H), 2.52 (br, 2 H, merged with residual H of DMSO-d_6), 1.78–1.71 (m, 2 H); ^{13}C NMR (100 MHz, DMSO-d_6): δ 202.5 (2 C), 102.8 (2 C), 94.9 (2 C), 80.4 (2 C), 73.8 (2 C), 51.9 (2 C), 51 (2 C), 39.5 (2 C) (merged with DMSO-d_6), 33.0 (2 C); HRMS (ESI): *m*/*z* $(M+H)^+$ calculated for $C_{18}H_{18}Cl_4O_8$: 502.9835; Found: 502.9837.

Bis-oxa-bridged derivative (10)

The reaction was performed as described for **8**; yield: 37%; colorless solid; mp: 230 °C (decomp.); IR (KBr): 2900, 1740, 1440, 1200 cm^{-1}; ^{1}H NMR (400 MHz, $CDCl_3$,): δ 3.82 (s, 6 H), 3.78 (s, 6 H), 3.39 (s, 6 H), 3.27 (s, 6 H), 3.03–3 (m, 2 H) 2.51 (dd, *J* = 11.7 Hz, 8.3 Hz, 2 H), 2.23 (dd, *J* = 11.7 Hz, 3.9 Hz, 2 H); ^{13}C NMR (100 MHz, $CDCl_3$): δ 166.8 (4 C), 110.1 (2 C), 92.5 (2 C), 88.7 (2 C), 52.6 (2 C), 52.4 (2 C), 51.9 (2 C), 51.6 (2 C), 41.8 (2 C), 34.8 (2 C); HRMS (ESI): *m*/*z* $(M+H)^+$ calculated for $C_{22}H_{30}O_{14}$: 519.1715; Found: 519.1717.

Preparation of bis-adducts 11–13

A mixture of 3-sulfolene (534 mg, 4.52 mmol), 1,2,3,4-tetrabromo-5,5-dimethoxycyclopentadiene (4 g, 9.05 mmol), hydroquinone (2 mg) and epichlorohydrin (0.1 mL) in benzene (2 mL) in a sealed tube was heated at 140–150 °C for 69 h. The volatiles were removed in vacuo and the products purified by silica gel column chromatography with 2–5% ethyl acetate/hexane as eluent to afford 105 mg of the mono adduct **11** (113 mg, 7%) as an *endo*:*exo* mixture (ratio 91:9) and the bis-adducts **12** and **13** as mixture of diastereomers in 1:1 ratio (3.2 g, 90%). The mixture **12** and **13** were quantitatively separated by preparative HPLC on a JAI LC-908W equipped with a JAIGEL-OA4100 column with 1% THF/hexane as eluent.

1,2,3,4-tetrabromo-7,7-dimethoxy-5-vinylbicyclo[2.2.1]hept-2-ene (11)

Colorless liquid; *endo*:*exo* = 91:9. IR (neat): 2900, 1630, 1430, 1240 cm^{-1}; ^{1}H NMR (400 MHz, $CDCl_3$,): *endo* (major isomer) δ 5.53–5.44 (m, 1H), 5.19–5.06 (m, 2H), 3.62 (s, 3 H), 3.57 (s, 3 H), 3.23 (td, *J* = 8.8 Hz, 4.1 Hz, 1 H), 2.54 (dd, *J* = 12.0 Hz, 8.8 Hz, 1H), 1.74 (dd, *J* = 12.0 Hz, 4.1 Hz, 1 H); *exo* (minor isomer) δ 5.99–5.90 (m, 1 H), 5.19–5.06 (m, 2 H, merged with major isomer), 3.62 (s, 3 H, merged with major isomer), 3.58 (s, 3 H), 2.39 (td, *J* = 8.8 Hz, 5.3 Hz, 1 H), 2.26–2.2 (m, 2 H); HRMS (ESI): *m*/*z* $(M+Na)^+$ calculated for $C_{11}H_{12}Br_4O_2$: 514.7469; Found: 514.7462.

1,1',4,4',5,5',6,6'-octabromo-7,7,7',7'-tetramethoxy-2,2'-bi(bicyclo[2.2.1]heptane)-5,5'-diene (12)

Colorless solid; mp: 196–198 °C. IR (KBr): 2850, 1580, 1430, 1240, cm^{-1}; ^{1}H NMR (400 MHz, $CDCl_3$): δ 3.57 (s, 6 H), 3.55 (s, 6 H), 2.62–2.59 (m, 2 H), 2.55–2.50 (m, 2 H), 2.44–2.43 (m, 2 H); ^{13}C NMR (100 MHz, $CDCl_3$): δ 127 (2 C), 123.8 (2 C), 110.6 (2 C), 73.2 (2 C), 67.5 (2 C), 53.3 (2 C), 51.8 (2 C), 48.9 (2 C), 44.1 (2 C); Anal. Calcd. for $C_{18}H_{18}Br_8O_4$: C 23.06, H 1.94; Found: C 23.31, H 1.90.

1,1',4,4',5,5',6,6'-octabromo-7,7,7',7'-tetramethoxy-2,2'-bi(bicyclo[2.2.1]heptane)-5,5'-diene (13)

Colorless solid; mp: 190–192 °C. IR (KBr): 2850, 1560, 1420, 1240 cm^{-1}; ^{1}H NMR (400 MHz, $CDCl_3$): δ 3.62 (6 H), 3.54 (s, 6 H), 3.05 (dd, J = 9.1 Hz, 3.9 Hz, 2 H), 2.42 (dd, J = 12.4 Hz, 9.1 Hz, 2 H), 1.44 (dd, J = 12.4 Hz, 3.9 Hz, 2 H); ^{13}C NMR (100 MHz, $CDCl_3$): δ 125.8 (2 C), 124.4 (2 C), 111.6 (2 C), 74.2 (2 C), 68.2 (2 C), 53.1 (2 C), 51.7 (2 C), 45.8 (2 C), 38 (2 C); Anal. Calcd. for $C_{18}H_{18}Br_8O_4$: C 23.06, H 1.94; Found: C 23.32, H 1.88.

Copies of ^{1}H and ^{13}C Spectra:

^{1}H NMR (400 MHz) and ^{13}C NMR (100 MHZ) of **4** in $CDCl_3$

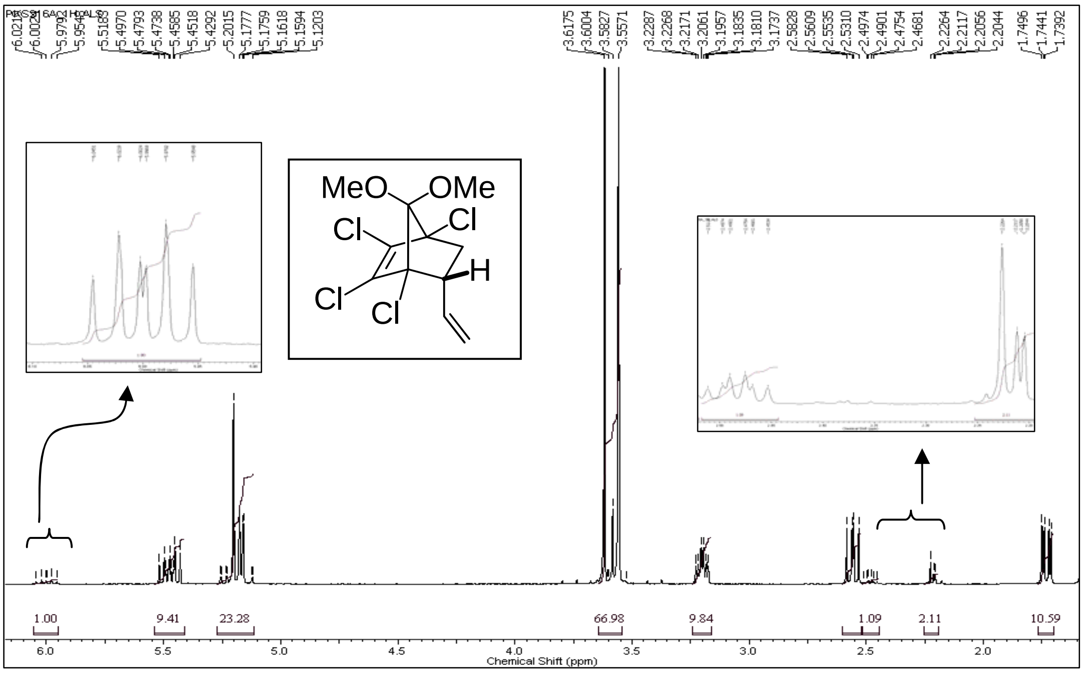

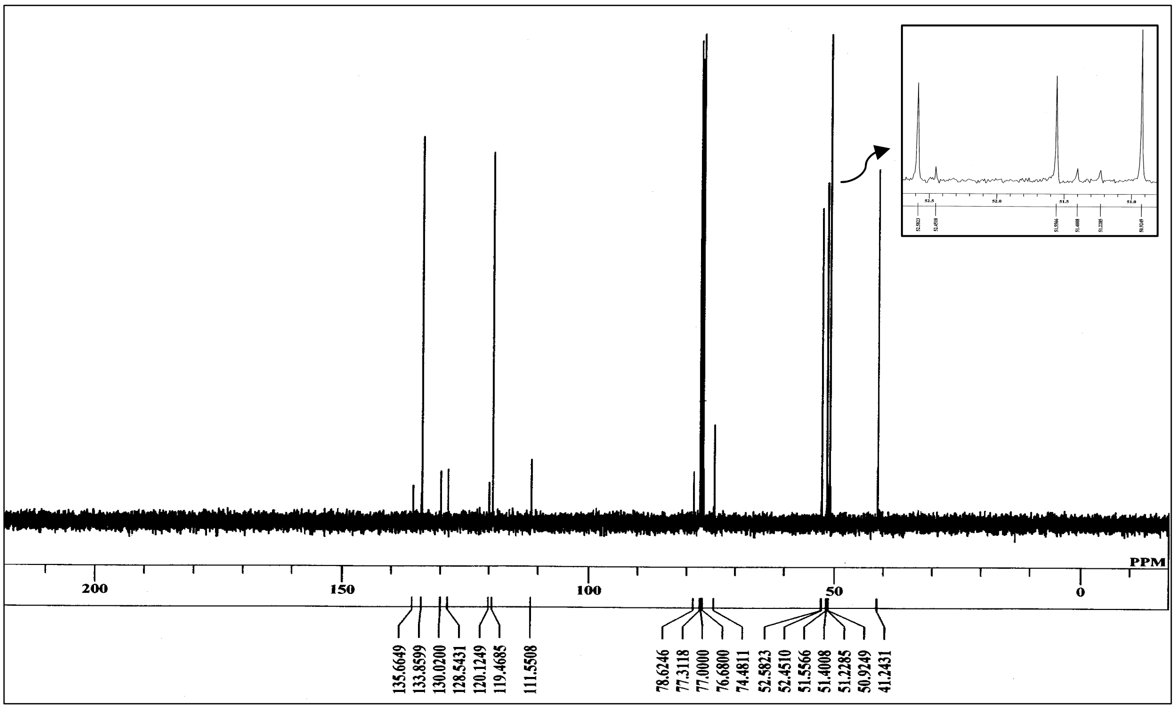

^{1}H NMR (400 MHz) and ^{13}C NMR (100 MHZ) of **5** in $CDCl_3$

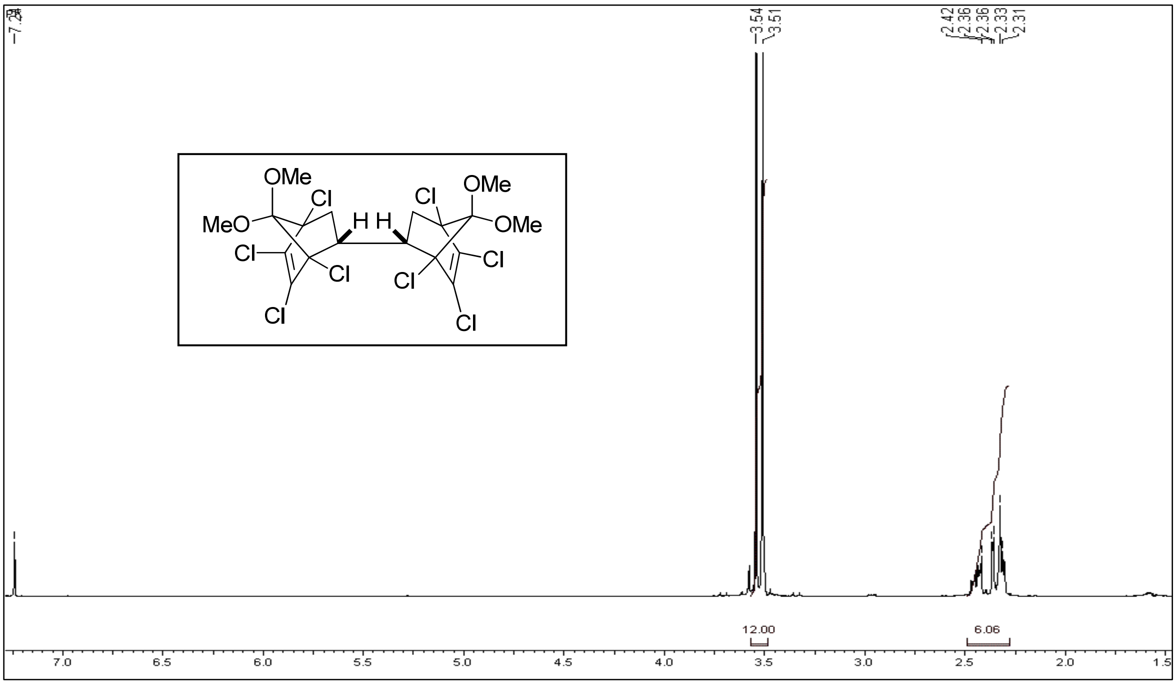

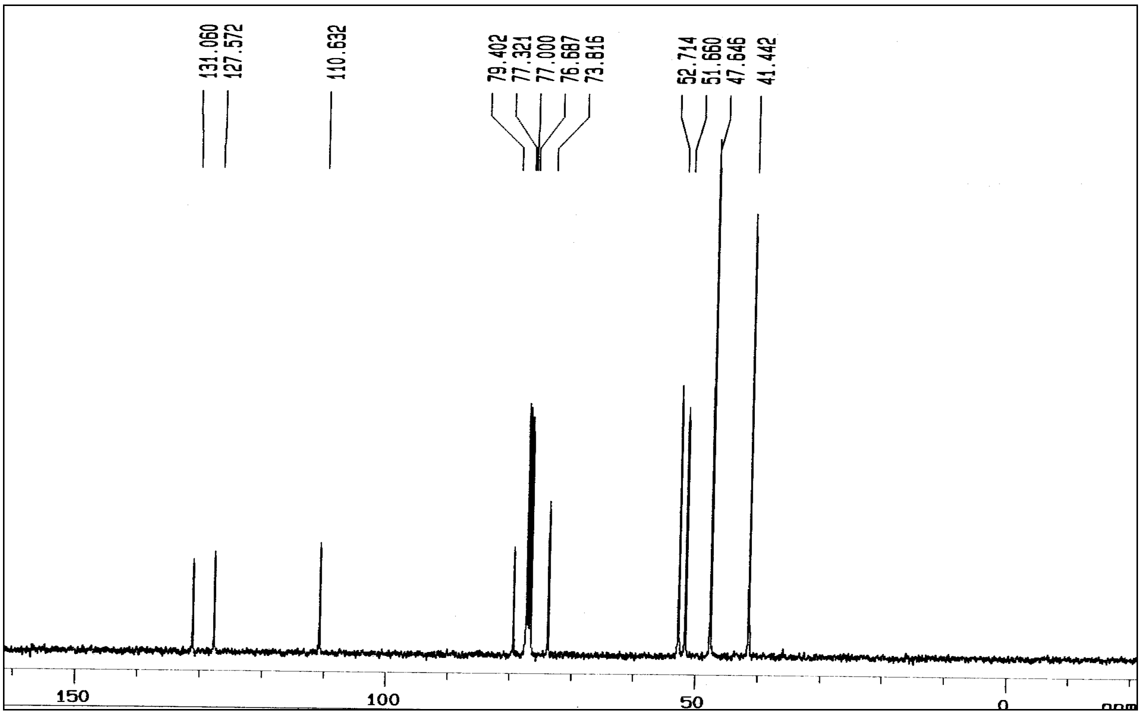

^{1}H NMR (400 MHz) and ^{13}C NMR (100 MHZ) of **6** in $CDCl_3$

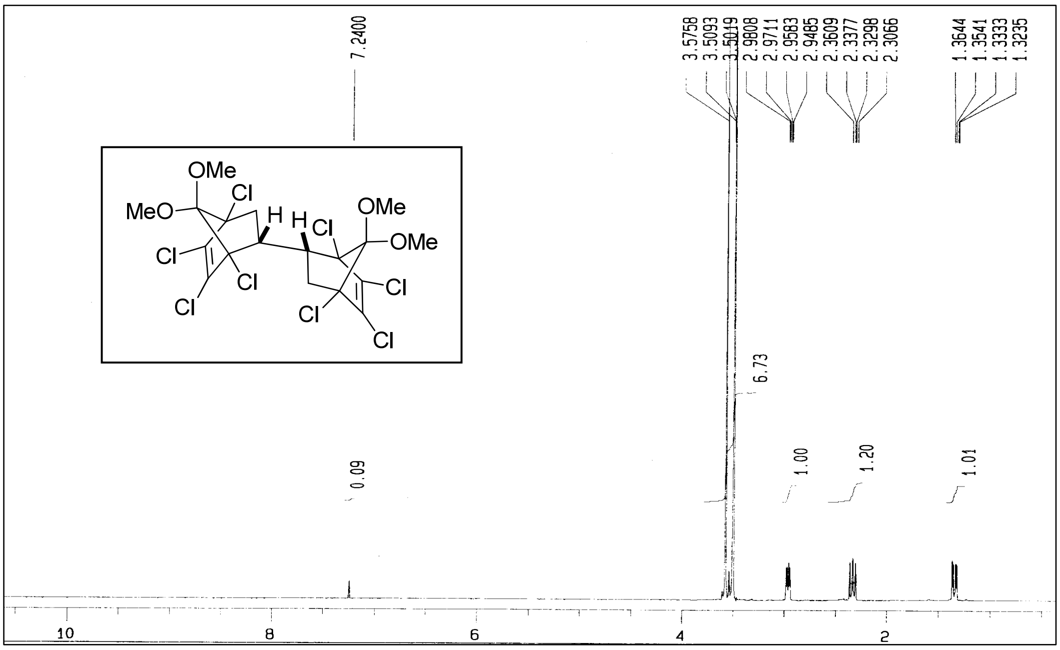

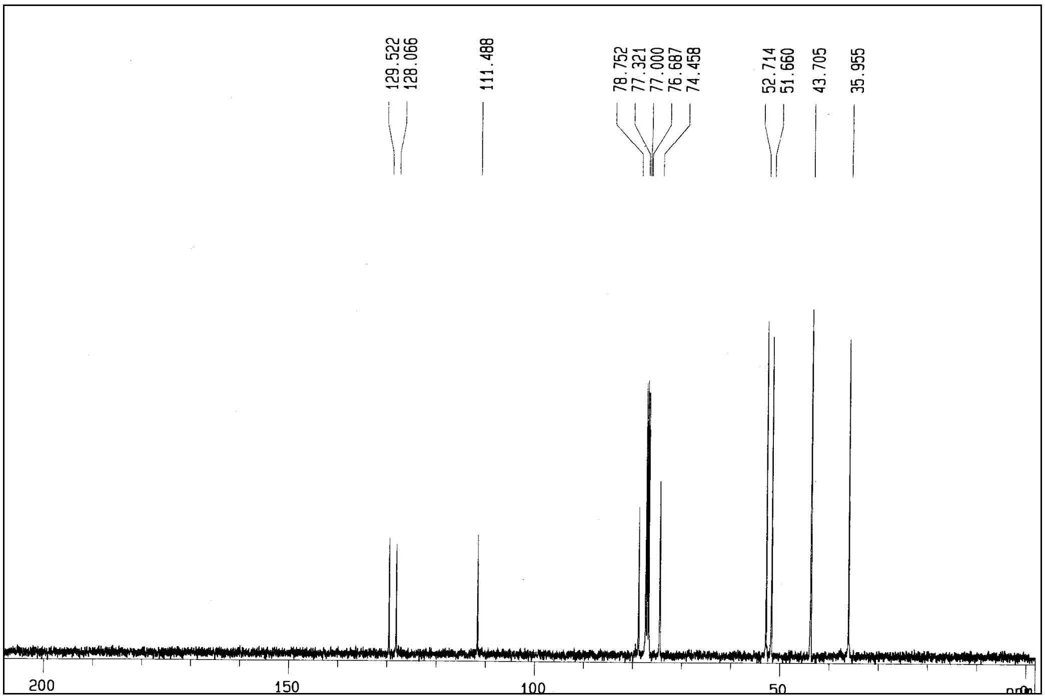

^{1}H NMR (400 MHz) and ^{13}C NMR (100 MHZ) of **7** in DMSO-d_6

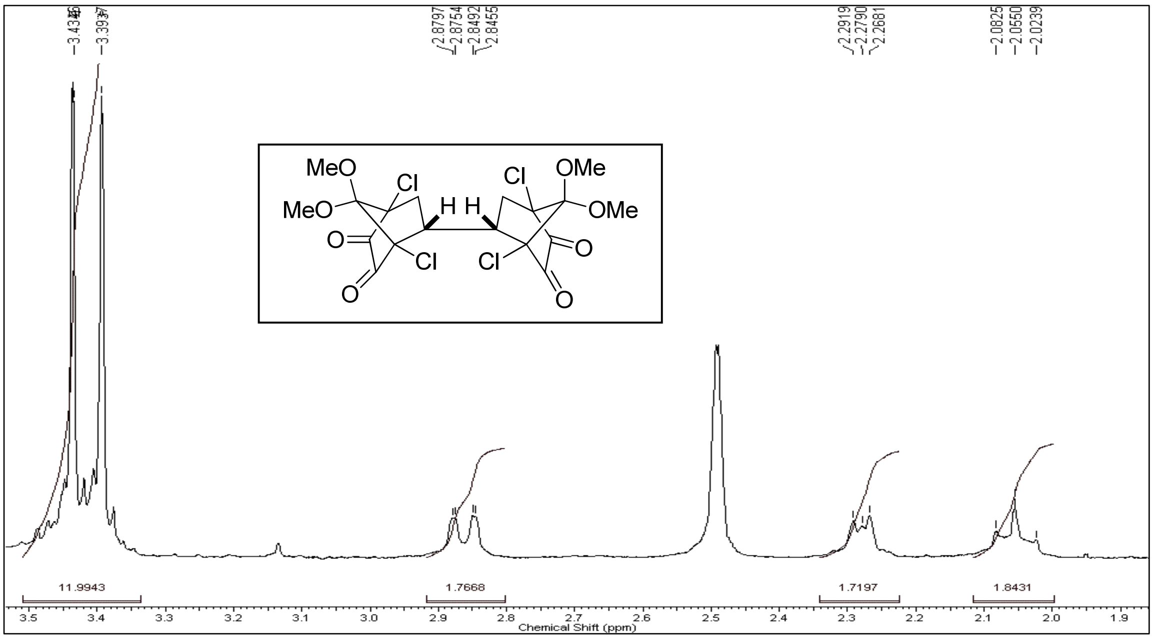

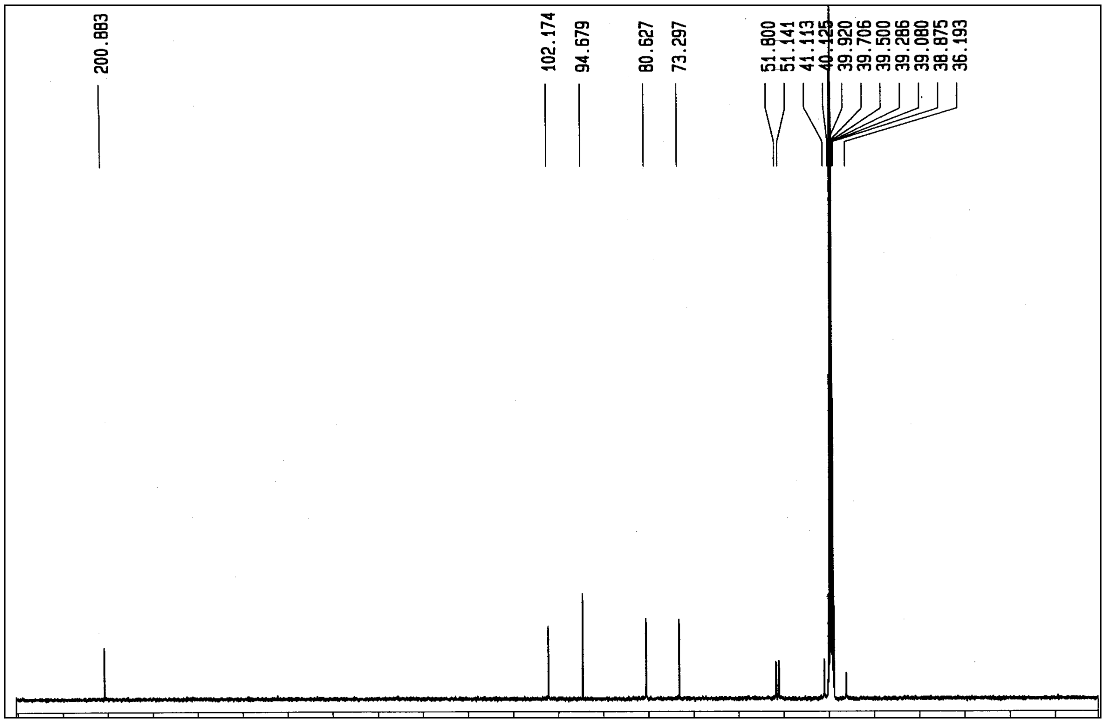

^{1}H NMR (400 MHz) and ^{13}C NMR (100 MHZ) of **8** in $CDCl_3$

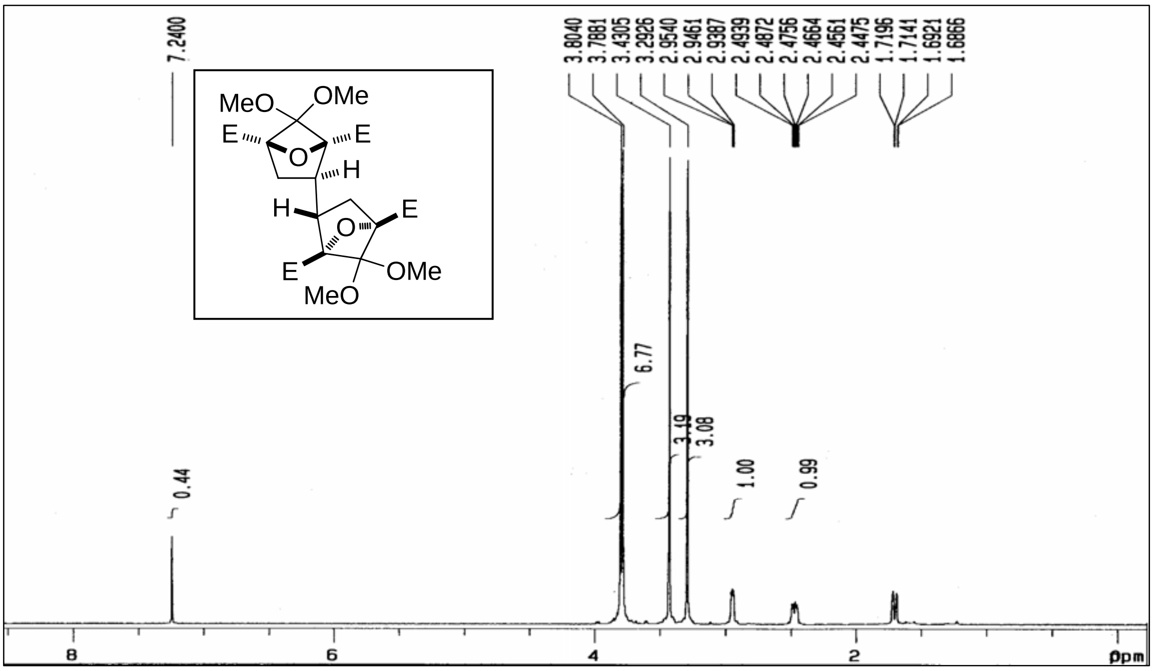

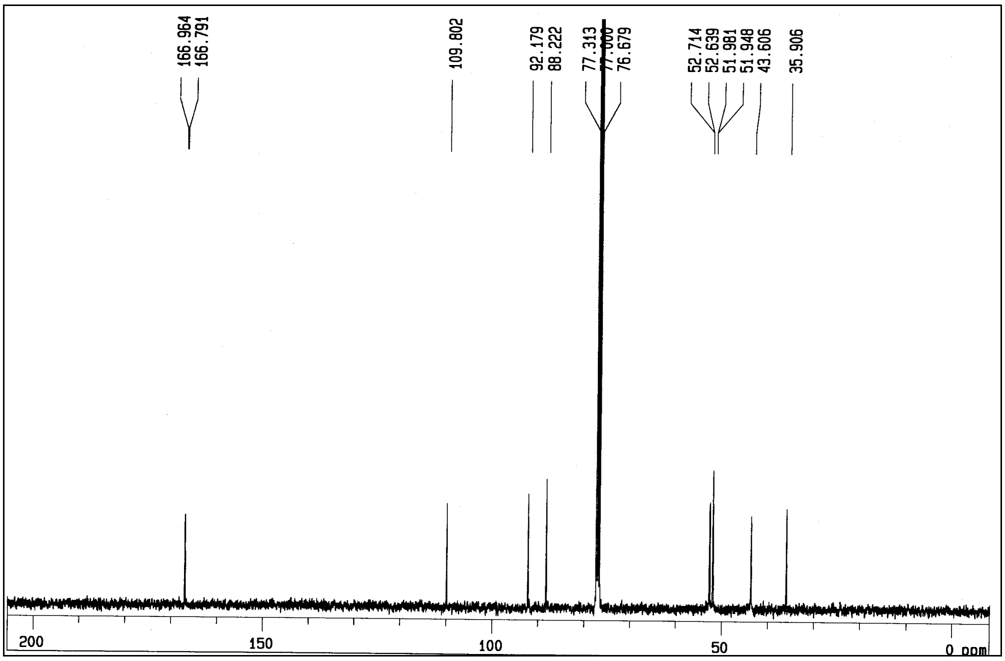

^{1}H NMR (400 MHz) and ^{13}C NMR (100 MHZ) of **9** in DMSO-d_6

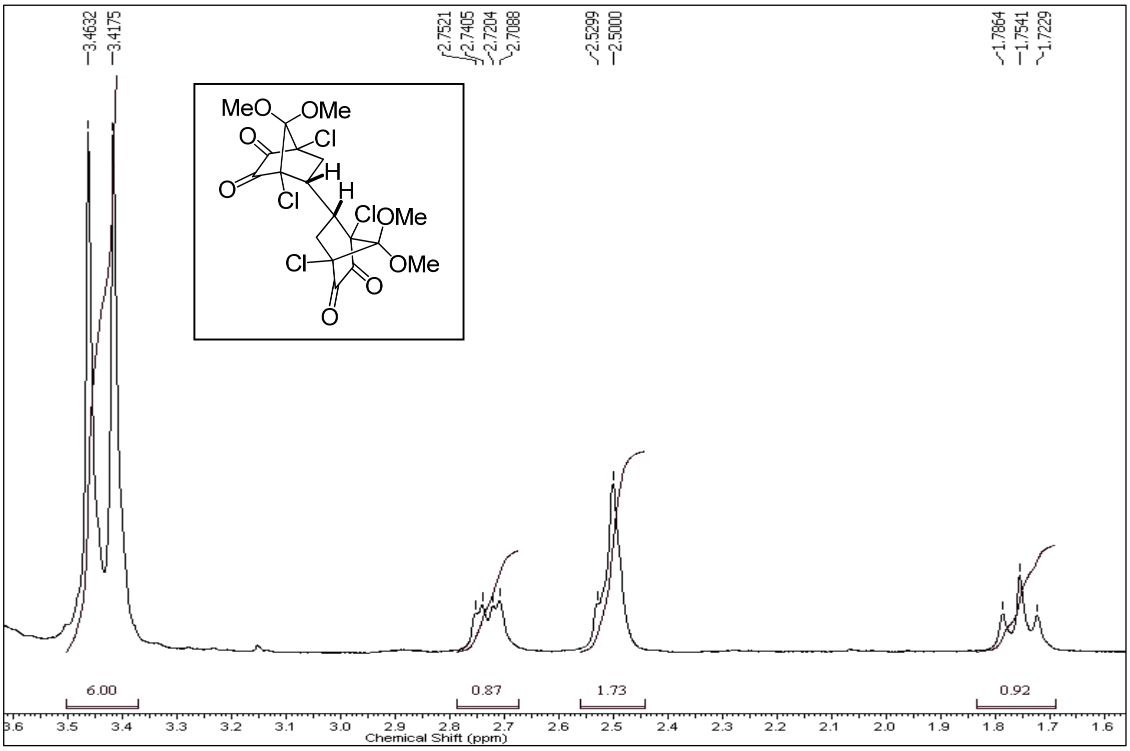

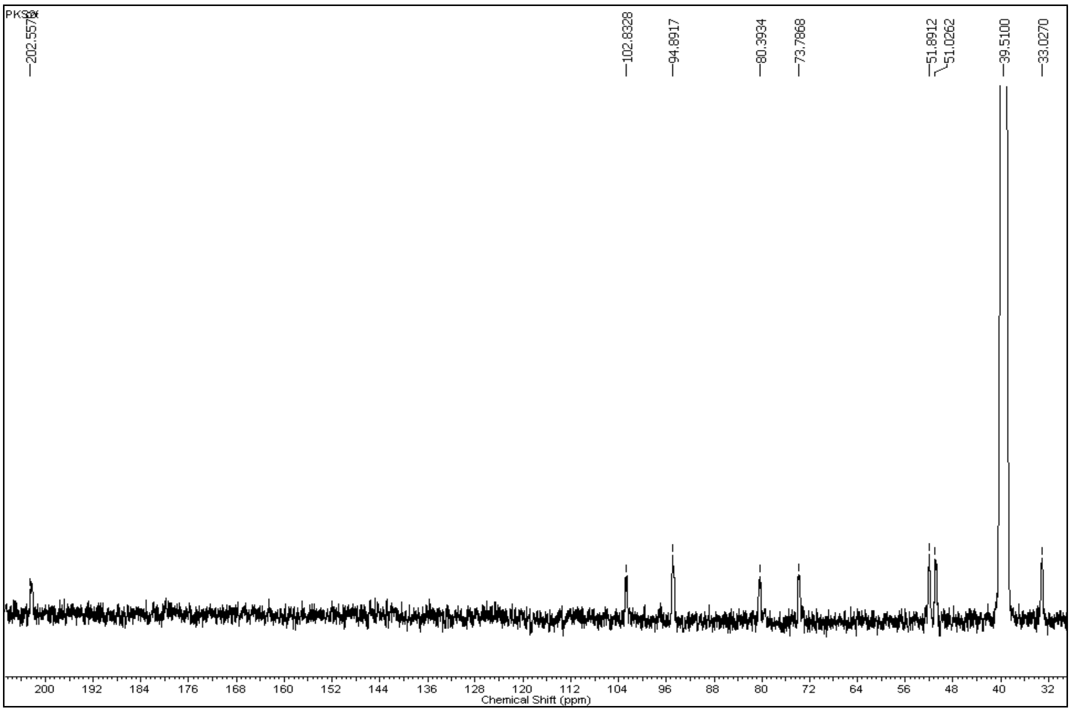

^{1}H NMR (400 MHz) and ^{13}C NMR (100 MHZ) of **10** in $CDCl_3$

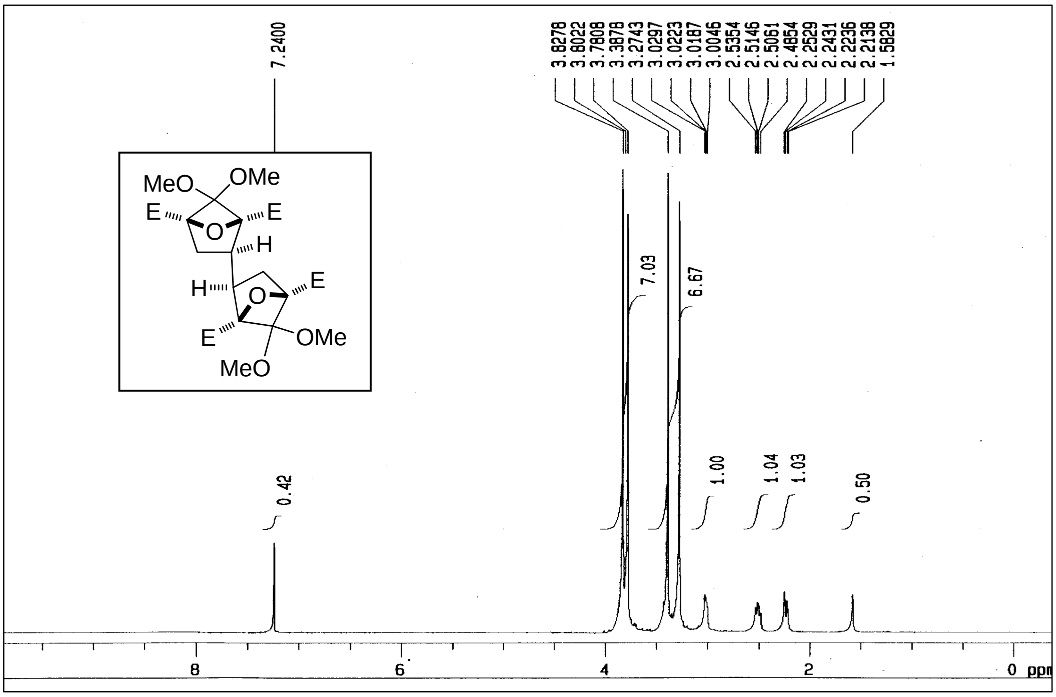

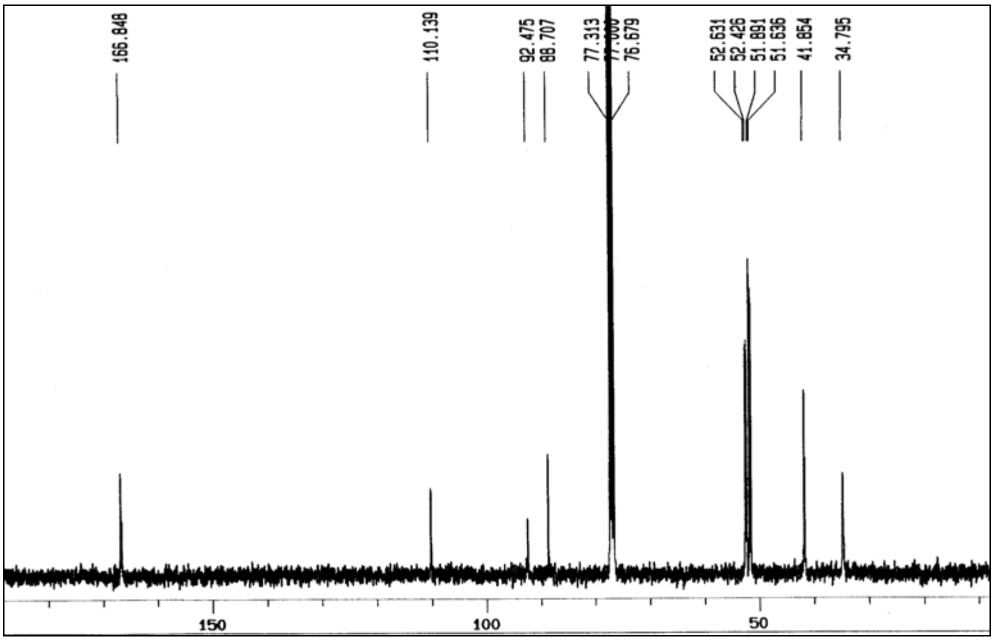

^{1}H NMR (400 MHz) of **11** in $CDCl_3$

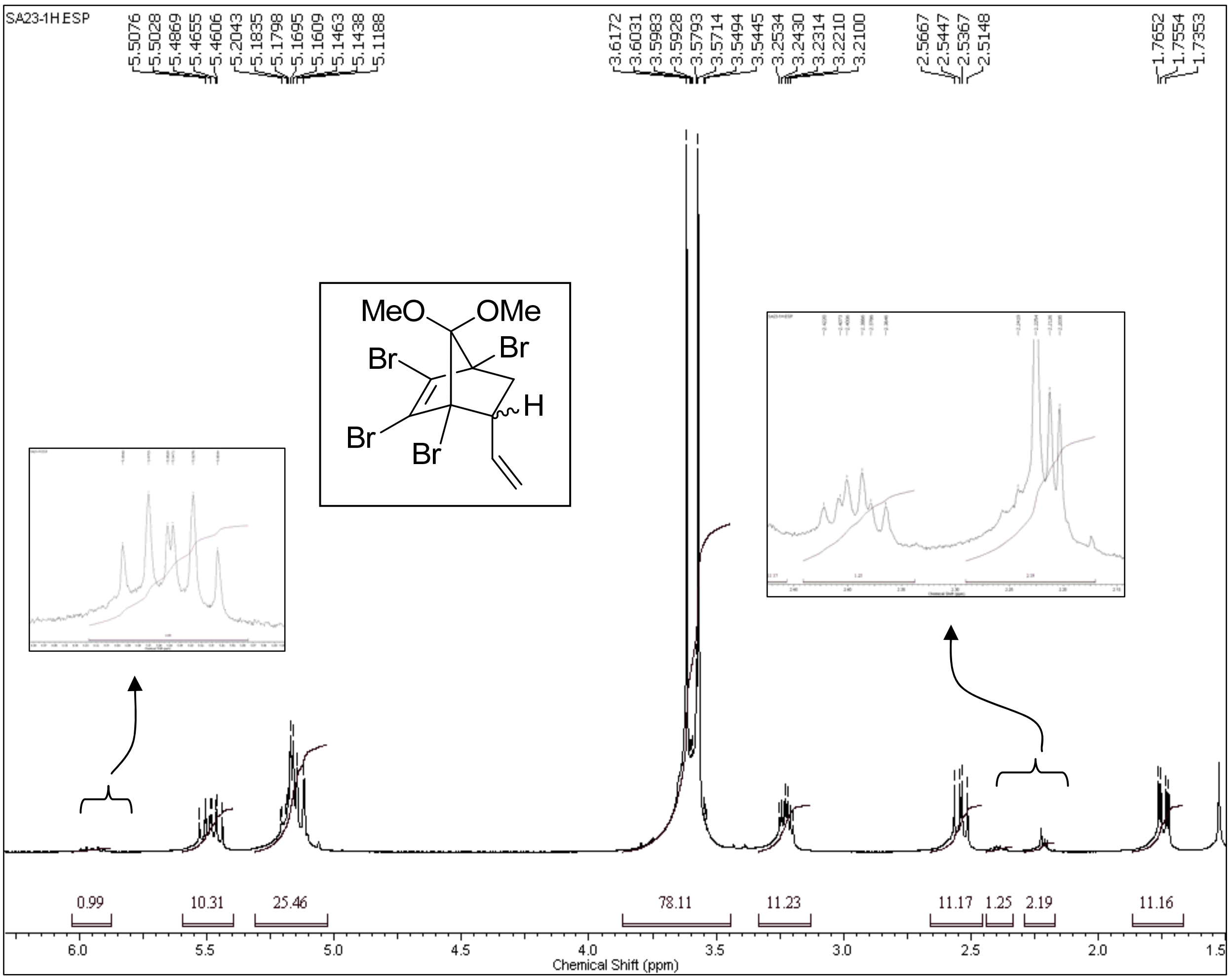

^1H NMR (400 MHz) and ^{13}C NMR (100 MHZ) of **12** in $CDCl_3$

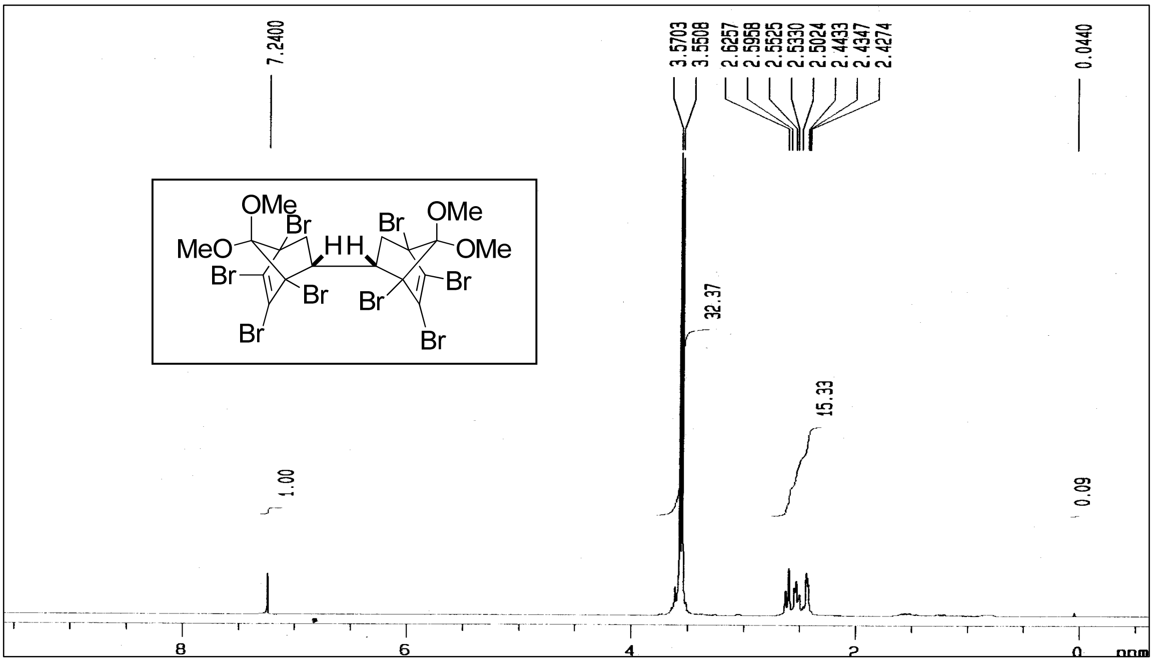

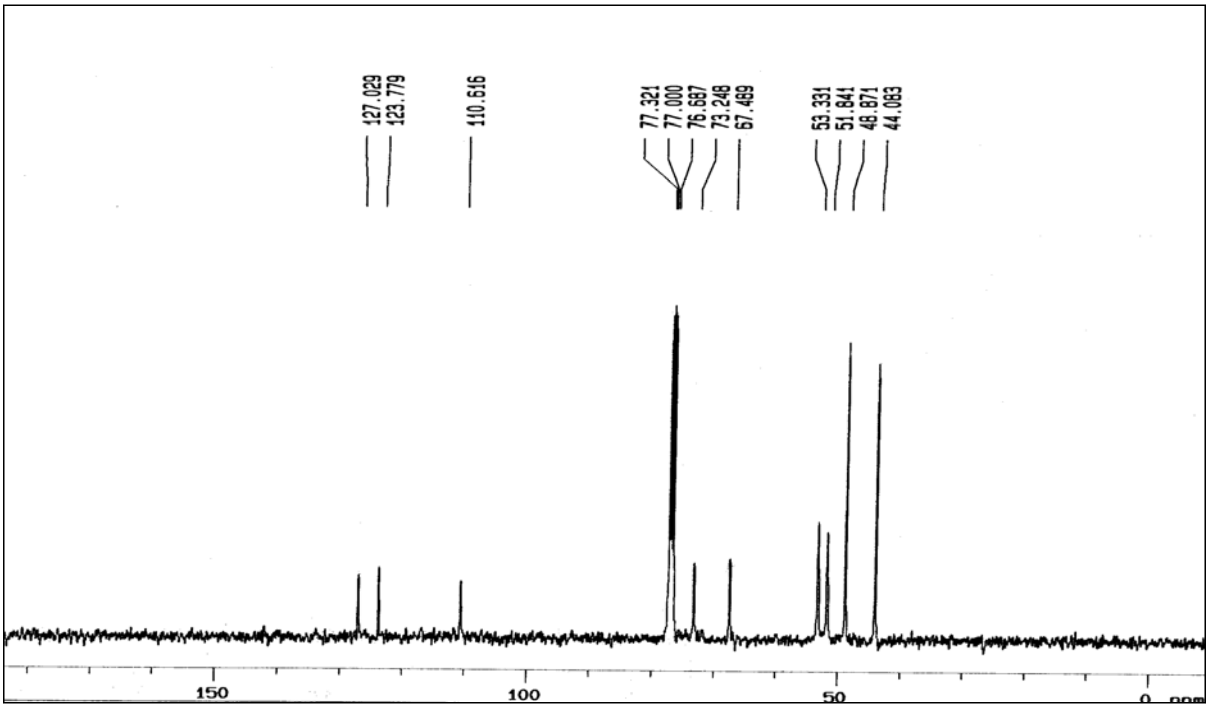

^{1}H NMR (400 MHz) and ^{13}C NMR (100 MHZ) of **13** in $CDCl_3$

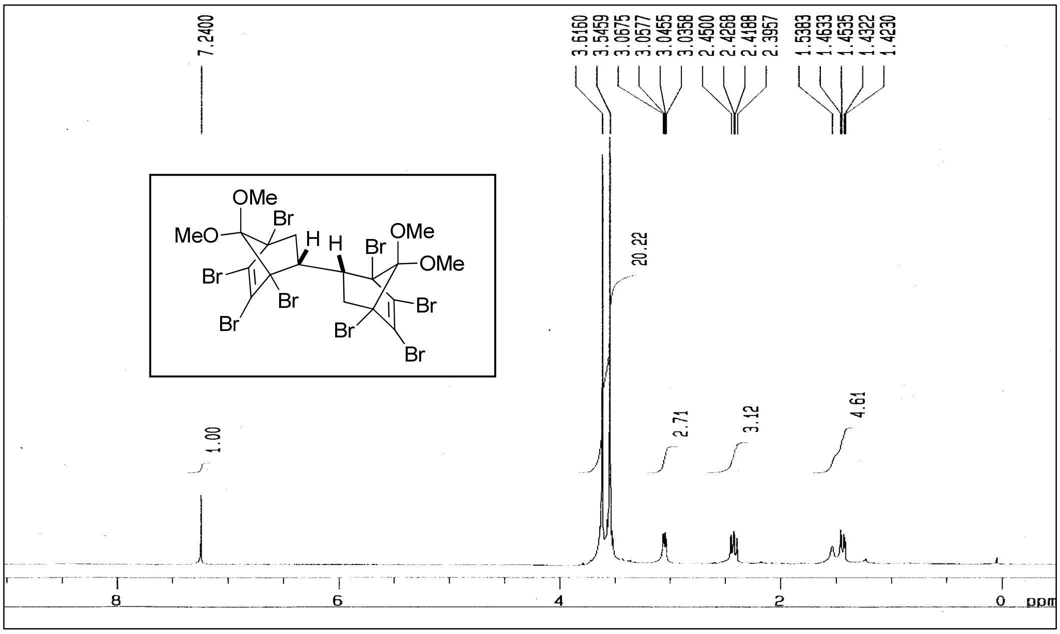

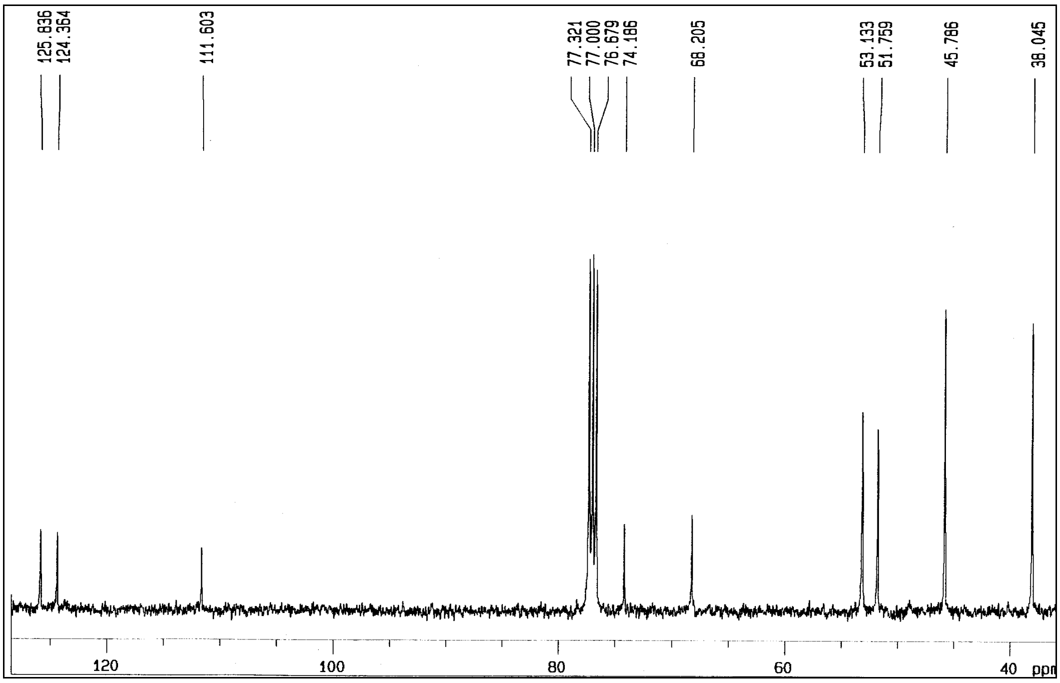

References

1. Perrin, D. D.; Armarego, W. L. F.; Perrin, D. R. *Purification of Laboratory Chemicals*, 2nd ed.; Pergamon Press: Oxford, 1980.
2. Sheldrick, G. M. SHELXL97, Program for the Refinement of Crystal Structures, University of Göttingen, Germany, 1997.
